# Suprarenal solitary fibrous tumor associated with a *NF1* gene mutation mimicking a kidney neoplasm: implications for surgical management

**DOI:** 10.1186/1477-7819-12-87

**Published:** 2014-04-07

**Authors:** Giovanni Conzo, Ernesto Tartaglia, Claudio Gambardella, Claudio Mauriello, Daniela Esposito, Massimo Mascolo, Daniela Russo, Gianfranca Stornaiuolo, Giovan Battista Gaeta, Luigi Santini

**Affiliations:** 1 Department of Anesthesiologic, Surgical and Emergency Science VII Division of General Surgery, Second University of Naples Italy, Via Pansini 5, 80131 Naples, Italy; 2 Department of Advanced Biomedical Sciences, University of Naples Federico II, Via Pansini 5, 80131 Naples, Italy; 3 Department of Infectious Diseases, Viral Hepatitis Unit, Second University of Naples, Via Pansini 5, 80131 Naples, Italy

**Keywords:** Solitary fibrous tumor, Spindle cells, Renal neoplasm, Immunohistochemical assay, Neurofibromatosis gene mutation

## Abstract

Solitary fibrous tumor (SFT) is a rare spindle cell neoplasm, usually occurring in the pleura. Pararenal SFT, mimicking an adrenal gland or renal tumor, as here described, is extremely rare. We report a case of a right suprarenal SFT, incidentally discovered by abdominal ultrasound in a 54-year-old woman carrying a point neurofibromatosis 1 (*NF1*) gene mutation. Preoperative diagnostic work-up was ineffective in evaluating its origin, and an open radical right nephrectomy was therefore undertaken. Immunohistochemical assay showed a positivity for CD34, CD99 and Bcl-2, so suggesting a diagnosis of SFT. According to our knowledge, the association between this type of tumor and *NF1* gene mutation has never been described. In cases of pararenal tumors, a more detailed preoperative diagnosis could be useful to better plan the extension of resection, allowing, in selected cases, nephron-sparing surgery. More studies are needed to better analyze the relationship between *NF1* gene mutation and SFT.

## Background

Solitary fibrous tumor (SFT) is a rare spindle cell neoplasm, firstly described in the1930s [1]. Prognosis seems to be favorable, but large series with long-term follow-up are still lacking. This kind of tumor, usually occurring in the pleura, the so called ‘localized fibrous mesothelioma’, has recently been described in different and multiple extrapleural sites including orbit, nasal cavity, breast, adrenal or thyroid gland, liver and lung [2]. Although the retroperitoneum is frequently involved, according to a computed search of the medical literature, only 39 cases of renal or pararenal SFT have been reported. Tumor size ranged from 2 to 25 cm, and nephrectomy was the standard treatment. The origin of these neoplasms is still controversial [3,4]. They are considered as slow-growing tumors with a favorable prognosis, although malignant cases, showing locoregional recurrence or distant metastases, have been reported. Neurofibromatosis type 1 (von Recklinghausen’s disease-*NF1*) is caused by an alteration of the *NF1* gene, a tumor suppressor located on the long arm of chromosome 17 (17q11.2) [5]. Loss of the gene function, due to a point mutation, leads to an increase in cell proliferation and to development of tumors [5].

We describe a right suprarenal SFT in a female patient carrying a *NF1* gene mutation. Preoperative diagnostic work-up was unable to determine its primal tissue, and therefore an open radical right nephrectomy was performed. Only the immunohistochemical assay allowed a correct pathological diagnosis. To better evaluate the role of radical nephrectomy, in a case of pararenal tumor with no clear signs of renal infiltration, more detailed therapeutic guidelines could be useful to establish the recommended extension of resection. According to our literature search, this is the first case in which SFT was associated to a *NF1* gene mutation.

## Case presentation

**Figure 1:**
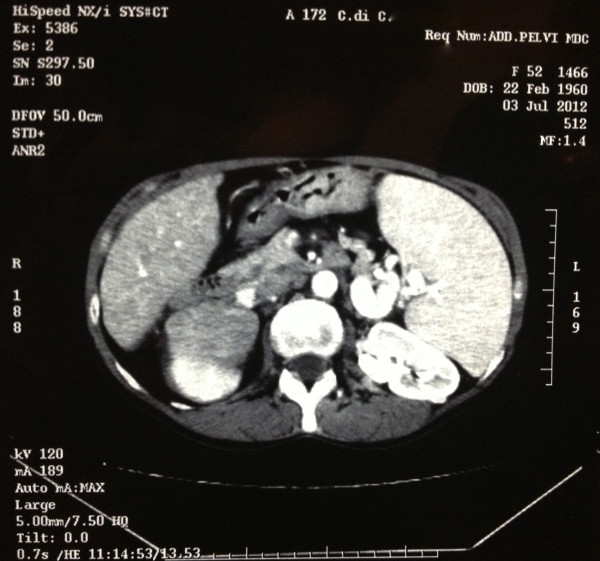
Computerized tomographic scan depicting large superior pole mass of the right kidney.

**Figure 2:**
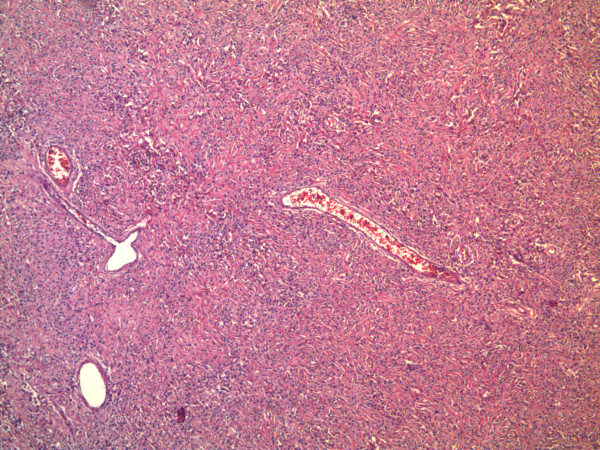
The tumor showed an admixture of irregularly distributed highly cellular and hypocellular zones, composed of spindled cells with vesicular nuclei, which surrounded thin- and thick-walled vessels with a characteristic ‘stag horn’ appearance.

**Figure 3:**
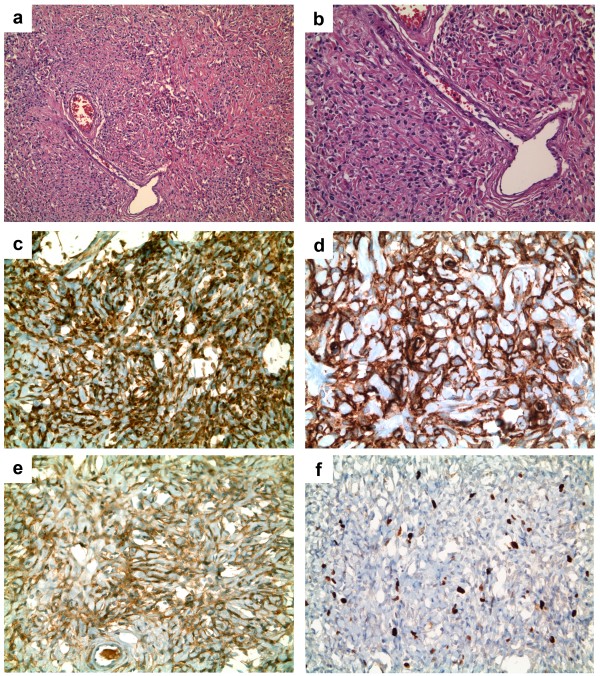
**A panoramic view of the solitary fibrous tumor. (a)** note the cellular proliferation of spindled cells, the dense collagen and the ‘stag horn’ vessels (H&E, x100); **(b)** The tumor cells surround a vascular component with a ‘stag horn’ appearance ((b) H&E, x150); **(c)** The tumor cells show a widespread immunoreactivity for CD34 (immunoperoxidase stain for CD34, x200); **(d)** The tumor cells exhibit a strong positivity for CD99 (immunoperoxidase stain for CD99, x200); **(e)** The tumor cells exhibit a diffuse staining for bcl-2 (immunoperoxidase stain for bcl-2, x200); **(f)** The tumor cells show a proliferation index to a maximum of about 10% of neoplastic cells (immunoperoxidase stain for Ki67/MIB-1, x150).

In July 2012, a 52-year-old woman was referred to our observation for the presence of a solid mass in her right kidney, detected during a routine follow-up for chronic hepatitis B virus infection. Computed tomography scan showed a well-delineated, encapsulated tumor arising from the upper pole of the right kidney and measuring 96 × 63 mm, contrast-enhanced and with no evidence of vessel infiltration (Figure 1). The ipsilateral adrenal gland was not clearly distinguishable from the neoplasm, and its adrenal origin was apparent. Magnetic resonance imaging (MRI) scan showed a mass of low intensity on T1-weighted images and of irregular high intensity on T2-weighed images. Neither renal vein nor inferior vena cava thrombosis were present, and suspected enlarged lymph nodes were also not identified. Due to the presence of a daughter with a previous diagnosis of *NF1* gene mutation, and the presence of small neurofibromas over the patient’s body, a genetic test for *NF1* using reverse transcription (RT)-PCR and high throughput-denaturing high performance liquid chromatography (HT-DHPLC) was performed, confirming the diagnosis of von Recklinghausen’s disease. Considering that a pheochromocytoma (PCC) has been identified in 0.1 to 5.7% of patients with von Recklinghausen’s disease, a PCC was considered in the differential diagnosis [6]. The absence of clinical signs and of arterial pressure lability, associated with a normal level of urinary metanephrine concentrations and the absence of pathological uptake following MIBG (meta iodo benzyl guanidine) scintigraphy, allowed the exclusion of a PCC neoplasm. Since a diagnosis of suspected renal or adrenal gland mass was suspected, the patient underwent an explorative laparotomy. During the procedure, a large neoplasm, strongly adherent to the upper renal pole and to the ipsilateral suprarenal space, was observed and, presuming a renal cell carcinoma, we undertook a surgical resection including a radical right nephrectomy. No macroscopic enlarged lymph nodes were observed. The postoperative course was uneventful and the patient was discharged on postoperative day four. Diagnostic work-up was negative during the eight-month follow-up. The gross specimen included: right kidney, ureter, adrenal gland, perinephric tissue and a mass measuring 90 × 60 × 40 mm in overall dimensions. At microscopy, the tumor showed an admixture of irregularly distributed highly cellular and hypocellular areas, composed of spindled cells with vesicular nuclei, which surrounded thin- and thick-walled vessels with a characteristic ‘stag horn’ appearance. A prominent hyalinized collagen was present (Figure 2). Most tumor cells expressed CD34, CD99, and bcl-2, whereas cytokeratin, CD117 (c-Kit), S-100 protein and EMA were negative. The Ki-67 (MIB-1) staining, a marker of cellular proliferation, was irregularly expressed, exhibiting positivity in up to about 10% of neoplastic cells. Based on morphological features and immunohistochemical profile, a diagnosis of a solitary fibrous tumor was made (Figure 3).

## Conclusions

In most cases of SFT, described as renal or pararenal tumor, it is very difficult to determine the true origin from the renal capsule [4], the interstitial tissue, or the peripelvic connective tissue. Therefore, in planning a therapeutic protocol, extension of resection may be controversial and ‘classical’ nephrectomy remains the most common treatment. In the reported case, only definitive pathology identified a well-encapsulated SFT not infiltrating the kidney and the ipsilateral adrenal gland, and radical nephrectomy could probably be considered an overtreatment. Histopathological examination, immunohistochemical and ultrastructural studies are the cornerstone of SFT diagnosis. Morphologically, it is characterized by spindle cell proliferation, and about 70% of cases expresses CD 34, CD99, and Bcl-2; only between 20 and 35% of cases are variably positive for epithelial membrane antigen and smooth muscle actin. Focal and limited reactivity of S-100 protein, cytokeratins and/or desmin has also occasionally been reported [6]. Strong CD34 reactivity is currently regarded as characteristic, and an indispensable finding in the diagnosis of SFT [7].

Since these tumors typically show hemangio-pericytomatous patterns, differential diagnosis includes sarcomatoid renal cell carcinoma or renal adenoma and other benign spindle cell tumors such as angiomyolipoma, fibroma, or fibrosarcoma [4]. In presence of a *NF1* gene mutation, neurofibroma should also be considered. SFT is a rare tumor sometimes mimicking renal cell or adrenal gland neoplasms [8,9], and it must be considered in cases of renal tumors comprising mesenchymal elements. In roughly 10 to 15% of cases, they behave aggressively, showing local recurrence or distant metastases [4]. According to England *et al*. [10], increased cellularity with crowded/overlapping nuclei, cellular pleomorphism, and a mitotic count of more than four figures per ten high-power fields are considered criteria for malignancy. However, clinical progression of tumors with a benign appearance cannot be predicted on histopathological basis, which can exhibit aggressive behavior, and *vice versa*. Therefore, all SFT patients need to be on long-term follow-up [4,7]. Turning to treatment, complete surgical resection is recommended [3], but because SFTs exhibit relative chemo-radio tumor resistance, no data are reported regarding the role of adjuvant treatment. In case of a preoperative suspicion of a well-encapsulated pararenal SFT, in the absence of clear signs of locoregional infiltration, the recommended extension of resection could be controversial. We hypothesize that, in selected cases, a nephrectomy could be avoided with comparable outcomes [11]. The rarity of pararenal SFT does not allow randomized prospective trials concerning this issue. Preoperative diagnostic efforts must be made to achieve detailed data about its origin in order to plan a tailored surgery. The association of STF and the *NF1* gene mutation is intriguing. However, since *NF1* gene mutation has not been investigated systematically in STF cases, and the available evidence in the literature data is insufficient, it is hard to hypothesize how a gene mutation might determine the development of a suprarenal neoplasm. More studies are needed to better analyze the relationships between SFT and *NF1* gene mutation.

### Consent

Written informed consent was obtained from the patient for the publication of this report and any accompanying images.

## Competing interests

The authors declare that they have no competing interests.

## Authors’ contributions

**CG:** Participated substantially in conception, design, and execution of the study; also participated substantially in the drafting and editing of the manuscript. **TE:** Participated substantially in conception, design, and execution of the study; also participated substantially in the drafting and editing of the manuscript. **GC:** Participated substantially in conception, design, and execution of the study. **MC:** Participated substantially in conception, design, and execution of the study. **ED:** Participated substantially in conception, design, and execution of the study. **MM:** Participated substantially in conception, design, and execution of the study. **RD:** Participated substantially in conception, design, and execution of the study. **SG:** Participated substantially in conception, design, and execution of the study. **GGB:** Participated substantially in conception, design, and execution of the study. **SL:** Participated substantially in conception, design, and execution of the study. All authors read and approved the final manuscript.
